# Modeling of the Electrotransport Process in PP-Based and PLA-Based Composite Fibers Filled with Carbon Nanofibers

**DOI:** 10.3390/polym14122362

**Published:** 2022-06-11

**Authors:** Olga Moskalyuk, Diana Vol‘nova, Ekaterina Tsobkallo

**Affiliations:** 1 Laboratory of Polymer and Composite Materials–SmartTextiles, IRC–X-ray Coherent Optics, Immanuel Kant Baltic Federal University, 236029 Kaliningrad, Russia; 2 Department of Engineering Materials Science and Metrology, St. Petersburg State University of Industrial Technology and Design, 191186 Saint-Petersburg, Russia; dianavolnova@yandex.ru (D.V.); tsobkallo@mail.ru (E.T.)

**Keywords:** fibers, polymer matrix, crystallinity, carbon nanofibers, electrotransport, modeling

## Abstract

Polypropylene and polylactide-based composite fibers have been produced by a melt technology. Long vapor-grown carbon fibers (CNFs) have been used as electrical conductivity fillers. It is clearly shown by experimental methods that the CNFs are evenly distributed in the polymer matrix, orienting themselves along the direction of fiber extrusion and retaining their initial dimensions. It is shown that for composites fibers based on crystallizing (polypropylene) and amorphous (polylactide acid) polymer matrix, the dependence of electrical resistance on the filler concentration is percolation character and can be described as a double Boltzmann function. Four sections are identified on the dependences of the electrical resistance on the filler concentration for composite fibers, and the reasons for this character of this dependence on the formation of electrically conductive circuits are analyzed. Investigated in this work are the PP-based and PLA-based composites filled with carbon nanofibers that can be used as antistatic, shielding materials, or as sensors.

## 1. Introduction

Polymer-based conductive composite materials are attracting great attention due to their unique characteristics, such as adjustable electrical conductivity, low specific gravity, and mechanical strength. These composites are widely used for the manufacture of electrical materials: sensors, antistatic and shielding materials, materials for energy, environmental and biomedical applications [1,2,3,4,5]. Conductive polymer composites are of particular interest in the creation of electrically conductive fibers/yarns for «smart» textile materials (e-textiles). The development of smart textiles has the potential to revolutionize the functionality of our clothing and the fabrics in our surroundings. Nanoscale manipulation results in new functionalities for intelligent textiles, including self-cleaning, sensing, actuating, and communicating [6,7]. The variety of fiber-forming matrices and conductive fillers used, the complexity and nonlinearity of the processes occurring during the formation of conductive clusters have led to the emergence of a scientific direction in the field of material science of chemical fibers, which studies the electrical properties of such composites [8,9,10,11,12].

The electrical conductivity of the polymer composite materials increases with a high content of electrically conductive filler. The dependence on the electrical resistivity of a composite based on an organic or inorganic matrix of filler concentration is presented in [13,14]. A feature of this dependence ρ (K) is an abrupt decrease in resistance at a high degree of filler severity caused by the transition of the insulator-conductor. The jump in the value of electrical resistivity, which can reach several orders of magnitude, is due to the formation of a continuous conductive chain of filler particles in the polymer matrix, called an infinite cluster.

It has been experimentally shown that the conductivity of composite fibers depends on a whole set of factors: the polymer matrix, the type of filler, its geometry and concentration, the degree of fiber drawing, manufacturing technology, etc. [15,16].

Currently, the process of electrotransport of composite materials is carried out in the field of scientific discussion. Some believe that charge transfer is carried out by conducting chains consisting of filler particles through direct contact. Other researchers believe that the conductivity of composite material is due to the thermal emission of electrons through the gaps between particles [17,18].

Of great interest is the possibility of predicting the volume of the percolation threshold without an experiment. Mathematical and computer modeling can be used for purposes of the processes taking place inside the composite. To date, many works in the literature are devoted to modeling the percolation process in general and in composite materials in particular.

The electrotransport process itself is modeled in the scientific literature in various ways. The influence of the concentration, the average size of the filler, and also its type on the electrical conductivity of polymer composites material was studied in [19]. It is shown that the main reason for the difference between the theoretical and experimental results on the prediction of the electrical conductivity of composite materials is the insufficiently complete consideration of the physical and chemical aspects that affect the flow of electric current in the material, in particular, such as the interfacial interaction between the polymer and nanoparticles.

In [20], the percolation process changes as the probability of a certain lattice, the nodes of which are fulfilled with a certain probability, which, in the general case, can depend on the parameters (time, position in the lattice). Simulation is carried out by the Monte Carlo method, which can be used for unfilled highly crystalline polymer matrix. However, the detection probability is calculated by the fitting method.

In [21], a linear dependence is used to describe the dependence of the electrical resistance on the filler concentration for polypropylene filled with acetylene black, graphite, aluminum powder, and zinc dust. This function cannot be used because it cannot describe the behavior of the function n (k) and does not provide an idea of the threshold value of the percolation threshold.

In [22], a model of the overall efficiency of the medium is used to simulate the electrically conductive properties of composite material. It has been shown that it allows predicting the electrical conductivity for different types of fillers at high loads. It has been established that the orientation of the composite material improves the mechanical characteristics while increasing the electrical conductivity of the composite.

Based on the interaction between filler particles, an analytical model was developed in [23] to predict the percolation threshold of conducting polymeric materials filled with graphite nanoplates and carbon nanotubes.

Computer simulation of the percolation process in homogeneous structures is presented in [24]. The percolation threshold was calculated using the Hoshen and Kopelman method of multiple labeling of clusters.

The paper [25] discusses in detail the question of how various sigmoidal models (S-models), such as Sigmoidal–Boltzmann (SB), Sigmoidal-Dose Response (SD), Sigmoidal–Hill (SH), Sigmoidal-Logistic (SL) and Sigmoid-Logistic-1 (SL-1) can be used to determine the percolation threshold of ethylene vinyl acetate (EVA) copolymer and acrylonitrile butadiene (NBR) copolymer of conductive composite systems filled with various carbon fillers.

Mathematical modeling of electrotransport processes makes it possible not only to carry out interpolation and, in certain cases, extrapolation of experimental data but also to understand the physical processes that occur during the formation of conducting clusters.

Of particular interest are composites with carbon fillers, in particular, with carbon nanofibers (CNF) [12]. Therefore, the study of the process of electrotransfer, the formation of conductive clusters in polymer matrices, especially in matrices in the form of oriented polymer structures (fibers) filled with various types of carbon nanoparticles, seems to be an important direction in the study of composite materials with special electrically conductive properties.

The aim of this work was to analyze and mathematical modeling of the concentration dependences of the specific volumetric electrical resistance of composite fibers obtained on the basis of fiber-forming polymer matrices differing in the ratio of amorphous and crystalline regions and filled with carbon nanofibers.

## 2. Materials and Methods

Composite fibers have been produced under laboratory conditions by melt spinning. Polypropylene (PP) Balen 01270 by Ufaorgsintez, Ufa, Russia, and polylactide (PLA) 2003D PLA by Nature Works, Minnetonka, Minnesota, USA, which is a mixture of 96% L-lactide and 4% D-lactide, were chosen as fiber-forming polymeric matrixes. A comparison of the structure and some of the properties of these fiber-forming polymers are described below and in Table 1.

Polypropylene (PP) is a high molecular weight thermoplastic polymer from the polyolefin class, obtained by polymerization of propylene at low pressure, and is partially crystalline and non-polar. Depending on the conditions of the polymerization process, polypropylene of various stereoisomeric compositions is obtained, but isotactic PP has found practical application. Isotactic PP belongs to the class of amorphous–crystalline polymers; that is, it spontaneously crystallizes at temperatures below the melting point of the crystalline phase. In such a polymorphic state, the volume of PP contains regions of various degrees of order, from completely disordered amorphous regions to highly ordered crystals. In amorphous regions, there is no three-dimensional periodic repetition of the structure; in crystalline regions, segments of macromolecules are arranged in a certain three-dimensional order. The degree of crystallinity of polypropylene can reach quite high values at the level of 70–85% [26,27].

Polylactic acid (PLA), also known as poly(lactic acid) or polylactide, is a thermoplastic polyester formally obtained by condensation of lactic acid with loss of water (hence its name) or by ring-opening polymerization of lactide, the cyclic dimer of the basic repeating unit. PLA has stereoisomers such as L-PLA, D-PLA, and LD-PLA. PLA with content of L-lactide above 90% crystallizes well, while a decrease in its amount leads to amorphization of the polymer [28,29].

Carbon nanofibers (CNFs, VGCF-H) manufactured by Showa Denko K.K. were used as a filler (Japan). CNFs are one of the allotropic forms of carbon with special properties, which are obtained by chemical vapor deposition. CNFs contain high-frequency graphite planes located around the circumference. CNFs are about 5 µm long and 150 nm in diameter. Unlike other carbon nanofillers, such as carbon black, multi-walled, and single-walled nanotubes, CNFs agglomerate weakly and have a fairly stable length. The electrical resistance of CNFs is 10^−6^ Ω·m.

The fillers have been dispersed in the polymer melt with a twin screw microcompounder DSM Xplore Microcompounder (The Netherlands). The melts were stirred for 5 min at 200 °C, and the screw rotation speed of 75 rev/min, and then the fibers were formed via a fixed circular die. Immediately after leaving the spinneret, the material was rapidly cooled by a flat jet of compressed air from the so-called “air knife” (pressure P = 3.9 Pa). The cooled material was wound at a constant speed receiver coil. As a result, the fibers containing different concentrations of nanofillers were obtained.

In previous work [30], we have shown that PP-based fibers obtained in this way have an amorphous–crystalline structure. Moreover, carbon particles act as nucleants in the crystallization processes of the fiber-forming matrix. In contrast, PLA-based composites, after extrusion, are in an amorphous state.

In order to obtain the values of the electrical resistivity of the fibers, the current-voltage characteristics (CVC) of the composites were measured using an automated CVC measurement system based on a Keithley 6487 picoammeter and an AKIP-1124 programmable DC power supply. To improve the contact between the sample and the electrodes, we used Conductive Carbon Paint (SPI) [12,15]. Volumetric electrical resistance was calculated by the formula:

$$\rho_{v}=\frac{U}{I}\times \frac{\pi \times D^{2}}{4L}$$

where *U* is the voltage measured from the rectilinear section of the CVC of the composite fibers, V; *I* is the current strength measured from the rectilinear section of the CVC of the composites, A; *D*—fibers diameter, m; *L* is the distance between the electrodes, m.

## 3. Results

**Figure 1:**
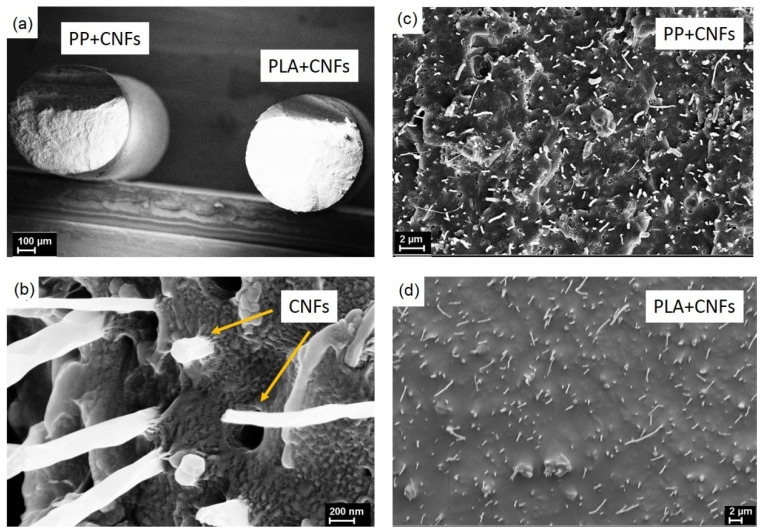
SEM images of the transversal cryo-cleaved of the PP-based and PLA-based composite fibers filled with 10 wt.% CNFs at different scales (**a**,**c**,**d**), of PP transcrystallites formed around the nanofillers (**b**).

The morphology of the nanocomposite polymer fibers was investigated by SEM (Figure 1) on the cryo-cleaved surfaces. Both composite fibers are characterized by a round cross section with a diameter of 400–500 μm (Figure 1a). One should note no particles of the CNFs were found on the natural fiber surfaces. For this reason, their distribution and interaction with the PLA and PP-matrix were evaluated on the cryo-cleaved surfaces of the composite samples. Figure 1c,d demonstrates the SEM images of the transverse cryo-cleavages of the fibers with 10 wt.% of CNFs. The remaining fibers were also investigated by SEM, but the major differences between the samples with different concentrations of the filler are only in the number of the visible nanoparticles.

SEM images illustrate a good dispersion of the nanoparticles of both polymer matrices. Typically, the CNFs were oriented along the fiber axis, although higher filler contents (>10 wt.%) led to some randomization of the orientations. The obvious fact must be noticed that all the CNFs serve as nucleation centers that facilitate the formation of PP transcrystallites on their surface (Figure 1b). The transcrystallites grow normally to the surface of the nanofillers.

**Figure 2:**
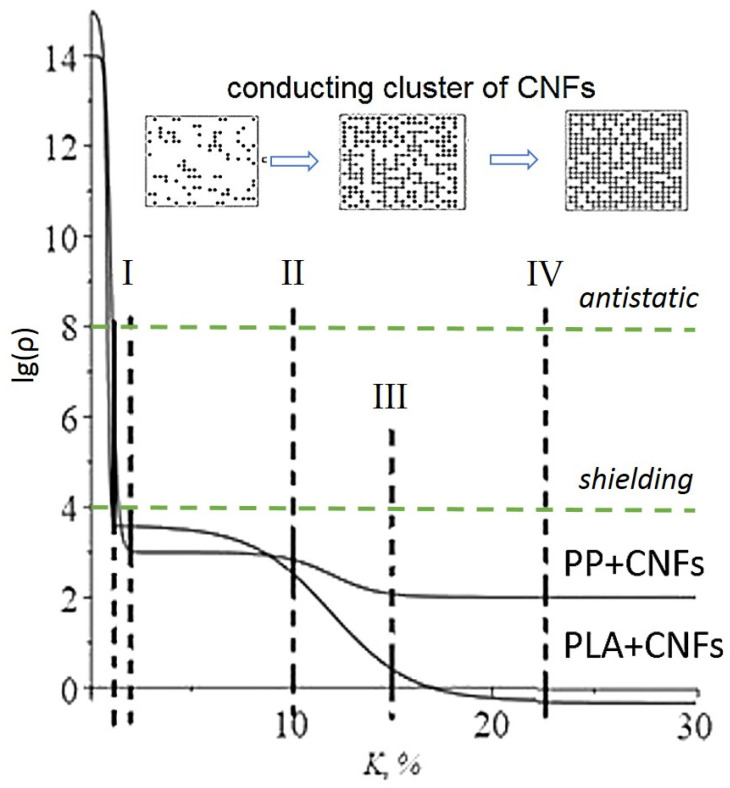
Dependences of the logarithm of electrical resistivity on the concentration of carbon nanofibers for composite fibers based on PP and PLA matrices: the black dotted lines separating the lg (ρ) dependences into sections (I–IV) characterize the different nature of the change in electrical resistance; green dotted lines characterize the upper limits of the electrical resistance values that provide the material with antistatic or shielding properties. The insert demonstrates the nature of the formation of a conducting cluster in a polymer matrix.

Experimental dependences characterizing the change in the specific volumetric electrical resistivity of PP-based and PLA-based composite fibers filled CNFs are shown in Figure 2 and Table 2. R = lg (ρ), ρ is the experimental value of volumetric electrical resistance, and K is filler concentration. The dependences (Figure 2) can be divided into four sections, which have a different character of the dependence lg(ρ): the first section (I)—K ∈ [0; 2.0] wt.% for PP-CNFs fibers, K ∈ [0; 1.1] wt.% for PLA-CNFs fibers; the second section (II)—K ∈ [2.1;10.0] wt.% for PP and K ∈ [1.2;10.0] wt.% for PLA; the third concentration site (III)—K ∈ [10.1; 15.0] wt.% for PP-CNFs fibers and K ∈ [10.1; 23.1] wt.% for PLA-CNFs fibers; the fourth section (IV)—K ∈ [15.1; +∞) wt.% for PP-CNFs fibers and K ∈ [23.2; +∞) wt.% for the PLA-CNFs fibers.

Green dotted lines characterize the upper limits of the electrical resistivity that provide the material with antistatic (ρv = 10^4^–10^8^ Ω·m) or shielding (ρv = 10^0^–10^4^ Ω·m) properties.

The insert (Figure 2, conducting cluster of CNFs) demonstrates the nature of the formation of a conducting cluster in a polymer matrix. With an increase in the concentration of the electrically conductive filler, the probability of the formation of new conductive chains increases. With the maximum introduction of the carbon filler, the electrically conductive network is formed throughout the entire volume of the polymer matrix, and no further increase in electrical conductivity is observed.

The analysis of the obtained results allows us to note the following dependencies features presented in Figure 2: R value process of changing has a clearly nonlinear form. In the area of filler concentration up to 1–2 wt.%, composite fibers have a level of electrical resistance sufficient for use as antistatic materials (ρv = 10^4^–10^8^ Ω·m); composites filled with a higher concentration of CNFs can be used for shielding and as sensors (ρv = 10^0^–10^4^ Ω·m) PLA-based composite fibers containing 20 wt.% CNFs are close in conductivity to conductive materials (ρv < 10^−1^ Ω·m) [31].

Dependence R(K) can be divided into four sections (Figure 2): two of which are characterized by a sharp decrease in the R value, and the R value on the other two is practically constant. Areas the specific volume resistance sharp change can be attributed to the course of percolation processes, which we will pay special attention to. In our works [11,13,14], the Boltzmann function (1) was used to model the concentration dependence of the specific volume electrical resistance in composite film filaments, which made it possible to describe the percolation process quite accurately.

(1) $$R=\frac{R_{1}-R_{2}}{1+e^{\frac{K-K_{0}}{△K}}}+R_{2}$$

where *R* = lgρ_v_, *R*_1_ = lgρ_1_—the electrical resistance initial value logarithm (i.e., the resistance of the polymer matrix); *R*_2_ = lgρ_2_—the electric resistance final value logarithm; *K*_0_—percolation threshold, i.e., the interval middle on which there is a sharp drop resistance, $\Delta K=\frac{R_{2}-R_{1}}{4R^{\prime }}$—value, determines the falling resistance speed at the filler concentration equal K_0_; the more Δ*K*, the less the tangent of the angle of inclination of the tangent to the graph of the function, i.e., slower falling resistance.

The Boltzmann function describes the percolation process well and explains the plateau. In future articles, we will present a more detailed substantiation of the choice of the Boltzmann function and an assessment of the adequacy of the chosen model for describing the electrotransport process in composite fibers.

Returning to the dependencies *R* = lg(ρ) obtained for the PP-CNFs and PLA-CNFs composites fibers, provided their nature (Figure 2) and the experience of modeling percolation «single» processes, it can be assumed that two functions superposition of the form (1) can be used to model complex percolation dependencies, i.e., the function of the form (2), which in the literature is called the double Boltzmann:

(2) $$R=\frac{R_{1}-R_{2}}{1+e^{\frac{K-K_{1}}{△K_{1}}}}+R_{2}\;-\;R_{3}1++R3$$

where *R*_1_ is the electrical resistance initial value of PP and PLA matrix; *R*_2_ is the electrical resistance value at which the first plateau appears; *R*_3_ is the electrical resistance value at which the second plateau appears; *K*_1_, *K*_2_ are the flow thresholds corresponding to each of the two «percolation» sections, Δ*K*_1_, Δ*K*_2_, determine the fall rates resistance values at the filler concentration *K*_1_ and *K*_2_, respectively.

The parameters of Equation (2) values for PP-based and PLA-based composite fibers are presented in Table 3.

Thus, SEM images illustrate a good dispersion of the CNFs of the PP and PLA matrix and are oriented along the fiber axis. CNFs serve as nucleation centers that facilitate the formation of transcrystallites on their surface. Dependences of the lg (ρ) on the concentration of carbon nanofibers for composite fibers based on PP and PLA matrices can be separated into four sections to characterize the different nature of the change in electrical resistance.

A Boltzmann function is proposed to describe the dependence of the electrical resistivity of the composite PP and PLA-based fibers filled with carbon nanofibers as nanofillers.

## 4. Discussion

Next, we will analyze the dependencies of R = lg (ρ) and compare the process of electrical transfer in PP-CNFs and PLA-CNFs composites fibers (Figure 2 and Table 3).

In the first section (I) (K ∈ [0; 2.0] wt.% for PP-CNFs fibers, K ∈ [0; 1.1] wt.% for PLA-CNFs fibers), the resistance drops very quickly, which indicates the formation of conductive chains of carbon nanofibers (clusters) already at the stage of spinning of fibers in both crystallizing (PP) and amorphous (PLA) matrices. However, there are also some differences, and it is appropriate to compare the values of R at a filler concentration of K = 1 wt.% for the two studied fibers (Table 3). It should be noted that in the PLA-CNFs fibers when spinning from a round die, the appearance of conductive paths occurs significantly more intensively, which can be explained by the absence of obstacles from the crystal structures, which at the first stage are areas of obstruction to the penetration of conductive carbon nanofibers and are absent in the amorphous PLA matrix.

It is also possible to note the dependence of the electrical resistance magnitude on the type of polymer matrix at the exit to the plateau (the end of the I section). For the PP-based composites fibers, when reaching the plateau, the value is R = 3.0; for the PLA-based composites fibers, this value is R = 3.6. The higher conductivity of PP-CNFs fibers at this stage can be explained by the following factors. At the first stage end, the formation of conductive chains, the presence of crystalline sites plays a positive role in the formation of clusters since they significantly reduce the amorphous polymer volume in which the conductive pathways are formed. The percolation threshold value and the speed of the process (i.e., the slope of the curve in the I section) are approximately the same for both matrices (Table 3).

The second section (II) is characterized by the output of the value to the plateau, which is observed for composite fibers formed based on both polymer matrices. The concentration boundaries of the plateau are approximately the same and do not depend much on the type of polymer matrix and amount to K ∈ [2.1;10.0] wt.% for PP and K ∈ [1.2;10.0] wt.% for PLA. The appearance of a plateau means that an increase in the filler concentration in this range may lead to the emergence of new conductive chains, but these chains may be longer, which is the reason for the insignificant contribution of these newly formed clusters to the reduction of resistance. Furthermore, an increase in the concentration in this range of values may lead to a certain reduction (optimization) of the lengths of the already existing conducting circuits—clusters [11,15,16].

At the third concentration site (III), which can be determined by the boundaries of the filler concentrations K ∈ [10.1; 15.0] wt.% for PP-CNFs fibers and K ∈ [10.1; 23.1] wt.% for PLA-CNFs fibers, a drop in resistivity is again observed, but at a significantly lower rate than on the first one. This may mean the formation of new conducting clusters and the healing of «gaps» in previously formed chains. The percolation threshold for both monofilaments is K_2_ = 12 wt.%, i.e., it does not depend on the polymer matrix; the process speed differs by 0.8 wt.%. This section of the CNFs concentration values is longer in the PLA-based fibers, which is explained by the large space for the formation of new conducting clusters in the amorphous phase with respect to the crystallizing polymer matrix. We note that the process of formation of conducting chains occurs only in amorphous regions.

The fourth section (IV), lying within K ∈ [15.1; +∞) wt.% for PP-CNFs fibers and K ∈ [23.2; +∞) wt.% for the PLA-CNFs fibers, shows the end of the process of formation of conductive structures. It should be noted that a further increase in concentration does not have the effect of reducing resistance. The final resistance of PP-based composite fibers is ρ= 100 Ω∙m; for PLA-based composite fibers is ρ= 0.6 Ω∙m. The resistivity of the CNFs is ρ = 10^−6^ Ω∙m. Thus, the electrical resistivity of the obtained fibers is 5–7 orders of magnitude higher (for PP and PLA, respectively) than the resistivity of the filler, which is typical for such composite structures.

Thus, it can be concluded б for composites fibers based on crystallizing, and for amorphous polymer matrices filled with carbon nanofibers, the dependence of electrical resistance on the filler concentration has a similar character and can be described by the double Boltzmann function (2). The appearance of conductive paths occurs significantly more intensively for PLA-CNFs fibers, which can be explained by the absence of obstacles from the crystal structures (PP-based fibers), which at the first stage are areas of obstruction to the penetration of conductive carbon nanofibers and are absent in the amorphous PLA matrix.

## 5. Conclusions

It is shown that the formation of PP-based and PLA-based composite fibers from the melt spinning using a round die contributes to the appearance of conductive chains even at low concentrations of anisotropic filler—carbon nanofibers. Some difference was noted in the formation of electrically conductive chains at the site of small concentrations of filler in fibers formed based on crystallizing and amorphous polymer matrices. For composites fibers based on crystallizing and amorphous polymer matrices and filled with carbon nanofibers, the dependence of electrical resistance on the filler concentration has a similar character and can be described by the double Boltzmann function (2). The appearance of conductive paths occurs significantly more intensively for PLA-CNFs fibers, which can be explained by the absence of obstacles from the crystal structures (PP-based fibers), which at the first stage are areas of obstruction to the penetration of conductive carbon nanofibers and are absent in the amorphous PLA matrix. The concentration boundaries of the plateau (II) are approximately the same and do not depend much on the type of polymer matrix. At the third concentration site (III), a drop in resistance is again observed, but at a significantly lower rate than on the first one. This may mean the formation of new conducting clusters and the healing of «gaps» in previously formed chains. The percolation threshold does not depend on the polymer matrix. This section of the CNFs concentration values is longer in the PLA-based fibers, which is explained by the large space for the formation of new conducting clusters in the amorphous phase with respect to the crystallizing polymer matrix. The fourth section (IV) shows the end of the process of formation of conductive structures, and a further increase in fillers concentration does not have the effect of reducing resistivity. The electrical resistivity of the obtained fibers is 5–7 orders of magnitude higher (for PP and PLA, respectively) than the resistivity of the filler, which is typical for such composite structures. In future articles, we will present a more detailed substantiation of the choice of the Boltzmann function and an assessment of the adequacy of the chosen model for describing the electrotransport process in composite fibers.

## Figures and Tables

**Table 1:** Structure and some of the properties of fiber-forming polymers.

Parameter	Polypropylene	Polylactic Acid
Backbone formula	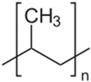	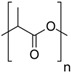
Density, g·cm^−3^	0.85–0.95	1.21–1.43
Volumetric electrical resistance, Ω·m	10^15^	10^14^

**Table 2:** R values corresponding to different concentrations of carbon nanofibers for PP-based and PLA-based composite fibers.

Samples	lg (ρ)						
	0 wt.%	1 wt.%	3 wt.%	5 wt.%	10 wt.%	15 wt.%	20 wt.%
PP-CNFs	15.0	8.8	3.1	3.1	2.5	2.0	2.0
PLA-CNFs	14.0	4.0	3.7	3.5	3.0	0.5	−0.3

**Table 3:** Values of function parameters (2) for PP-based and PLA-based composite fibers filled with CNFs.

Function Parameters (2)	PP-CNFs	PLA-CNFs
*R*_1_	15.0	14.0
*R*_2_	3.0	3.6
*R*_3_	2.0	−0.3
*K*_1_	1.0	0.8
*K*_2_	12.0	12.0
Δ*K*_1_	0.16	0.06
Δ*K*_2_	1.20	2.00

## Data Availability

Not applicable.
